# MMP-3 Contributes to Nigrostriatal Dopaminergic Neuronal Loss, BBB Damage, and Neuroinflammation in an MPTP Mouse Model of Parkinson's Disease

**DOI:** 10.1155/2013/370526

**Published:** 2013-06-19

**Authors:** Young Cheul Chung, Yoon-Seong Kim, Eugene Bok, Tae Young Yune, Sungho Maeng, Byung Kwan Jin

**Affiliations:** ^1^Department of Biochemistry and Molecular Biology, School of Medicine, Kyung Hee University, Seoul 130-701, Republic of Korea; ^2^Neurodegeneration Control Research Center, School of Medicine, Kyung Hee University, Seoul 130-701, Republic of Korea; ^3^Laboratory of Neurobiology and Genetics, The Rockefeller University, New York, NY 10065, USA; ^4^Burnett School of Biomedical Sciences, College of Medicine, University of Central Florida, Orlando, FL 32827, USA; ^5^Aged-Related and Brain Disease Research Center, School of Medicine, Kyung Hee University, Seoul 130-701, Republic of Korea; ^6^Department of East-West Medicine, Graduate School of East-West Medical Science, Kyung Hee University, Yongin 446-701, Republic of Korea

## Abstract

The present study examined whether matrix metalloproteinase-3 (MMP-3) participates in the loss of dopaminergic (DA) neurons in the nigrostriatal pathway in a 1-methyl-4-phenyl-1,2,3,6-tetrahydropyridine (MPTP) mouse model of Parkinson's disease with blood brain barrier (BBB) damage and infiltration of peripheral immune cells. Tyrosine hydroxylase (TH) immunostaining of brain sections from MPTP-treated mice showed that MPTP induced significant degeneration of nigrostriatal DA neurons. Moreover, FITC-labeled albumin detection and immunostaining revealed that MPTP caused damage to the BBB and increased the number of ED-1- and CD-3-immunopositive cells in the substantia nigra (SN). Genetic ablation of MMP-3 reduced the nigrostriatal DA neuron loss and improved motor function. This neuroprotective effect afforded by MMP-3 deletion was associated with the suppression of BBB disruption and a decrease in the number of ED-1- and CD-3-immunopositive cells in the SN. These data suggest that MMP-3 could play a crucial role in neurodegenerative diseases such as PD in which BBB damage and neuroinflammation are implicated.

## 1. Introduction

Parkinson's disease (PD) is a common neurodegenerative disease associated with progressive degeneration of the nigrostriatal dopaminergic (DA) pathway [1]. Although the etiology of PD and the mechanisms that mediate disease development remain largely unknown, accumulating clinical and experimental evidence suggests that PD is associated with neuroinflammatory processes such as microglial activation, T-leukocyte infiltration, and blood brain barrier (BBB) dysfunction [2–4]. Microglial and/or macrophage phagocytotic activity [3] and T-leukocyte infiltration [5] are upregulated in damaged areas of the midbrain of PD patients and in the brains of MPTP-treated mice, thus giving rise to the death of DA neurons. Increased BBB permeability [6] and blood vessel changes [7] have also been reported in PD patients and similarly contribute to DA neuronal death in MPTP-treated animal models of PD [8, 9].

Matrix metalloproteinase-3 (MMP-3) is a zinc-dependent proteolytic enzyme that is converted to active MMP-3 through autocleavage; the active form remodels the extracellular matrix complex (EMC) in the basal lamina which forms part of the BBB [10]. Besides degradation of ECM molecules, MMP-3 can activate pro-MMPs (pro-MMP-1, -3, -7, -8, -9, and -13) and cleave cell adhesion molecules, chemokines, and cytokines [11]. The widespread distribution of MMP-3 in the brain suggests that it plays a crucial role in the central nervous system (CNS). MMP-3 is involved in axonal growth, neuronal migration and synaptogenesis in brain development [12, 13], and in synaptic plasticity in learning and memory [14]. In contrast to these functions, it has been shown that MMP-3 released from apoptotic neuronal cells causes microglia activation and increases inflammatory processes *in vitro* [15]. In addition, MMP-3 facilitates BBB disruption and neutrophil influx in the cerebral cortex of LPS-injected mice as a consequence of its action on the basal lamina and tight junction proteins [16].

Increasing evidence suggests that MMP-3 plays an important role in the pathogenesis of neurodegenerative diseases such as Alzheimer's disease, vascular dementia, ischemic stroke, and PD [10]. In particular, several *in vitro* studies reported that MMP-3 exerted neurotoxic effects on oxidative stress- and endoplasmic reticulum stress-triggered DA neuronal death through caspase-3 activation [17, 18]. Moreover, MMP-3 immunoreactivity was elevated in the SN of 6-hydroxydopamine-(6-OHDA-) injected rats [19] and colocalized within Lewy bodies in the postmortem brains of PD patients [20]. Under the neuropathological conditions of PD, MMP-3 participates in DA neuronal cell death through the proteolytic cleavage of *α*-synuclein *in vivo* and *in vitro* [20]. In addition, ghrelin and exendin-4 have been shown to attenuate nigrostriatal DA neuronal loss and microglial activation via inhibition of MMP-3 expression in the MPTP mouse model of PD [21, 22]. However, in the context of PD, little is known about the role of MMP-3 in relation to BBB function and the infiltration of peripheral immune cells in the nigrostriatal DA system. In the current study, therefore, we have used the MPTP mouse model of PD with a view to determining if MMP-3 exacerbates the degeneration of nigrostriatal DA neurons as a consequence of its action to disrupt the BBB and allow the infiltration of T leukocytes into the brain.

## 2. Materials and Methods

### 2.1. Animals and Treatment

Eight-to nine-week-old male C57BL/6J mice (MMP^+/+^; Jackson Laboratory) and C57BL/6J-inbred mice deficient for matrix metalloproteinase-e (*MMP-3* ^−/−^) were used [23]. All experiments were performed in accordance with the approved animal protocols and guidelines established by Kyung Hee University (KHUASP(SE)-10-030). For MPTP intoxication, mice received four intraperitoneal injections of MPTP (20 mg/kg, free base; Sigma) dissolved in saline at 2 h intervals by following the previously reported method [24, 25].

### 2.2. Tissue Preparation and Immunohistochemistry

Mice brain tissues were prepared for immunostaining as described previously [24–26]. Briefly, brain sections were rinsed in PBS and incubated overnight at room temperature with primary antibodies. The primary antibodies included those directed against tyrosine hydroxylase (TH, 1 : 2000 dilution; Pel-Freez Biologicals, Rogers, AR, USA) for DA neurons, ED-1 (CD68, 1 : 1000 dilution; Serotec, Oxford, UK) for phagocytotic microglia/macrophage, and CD-3 (1 : 500 dilution; Serotec, Oxford, UK) for T leukocytes. The following day, sections were rinsed with PBS and 0.5% bovine serum albumin (BSA), incubated with the appropriate biotinylated secondary antibody, and processed with an Avidin-Biotin Complex Kit (Vectastain ABC kit; Vector Laboratories, Burlingame, CA, USA). Bound antiserum was visualized by treatment with 0.05% diaminobenzidine-HCl (DAB) and 0.003% hydrogen peroxide in 0.1 M PB. The DAB reaction was terminated by rinsing tissues in 0.1 M PB. Labeled tissue sections were mounted on gelatin-coated slides and analyzed under a bright-field microscope (Nikon, Melville, NY, USA). 

### 2.3. Stereological Cell Counts

 The unbiased stereological estimation of the total number of the TH-ip neurons was made using the optical fractionator method performed on an Olympus Computer-Assisted Stereological Toolbox (CAST) system version 2.1.4 (Olympus Denmark A/S, Ballerup, Denmark) in the various animal groups at 7 days postinjection (MPTP or saline) as previously described [25, 27]. Actual counting was performed using a 100x oil objective. The total number of neurons was estimated according to the Optical Fractionator Equation [28]. More than 300 points over all sections of each specimen were analyzed.

### 2.4. Densitometric Analysis

 As previously described [25, 27], the optical density of TH-positive fiber in STR was examined at 5x magnification using the Image Pro Plus system (Version 4.0, Media Cybernetics, Silver Spring, Maryland, USA) on a computer attached to a light microscope (Zeiss Axioskop, Oberkochen, Germany) interfaced with a CCD video camera (Kodak Mega Plus model 1.4 I, New York, NY, USA). To determine the density of the TH staining in the STR, a square frame of 700 × 700 mm was placed in the dorsal part of the STR. A second square frame of 200 × 200 mm was placed in the region of the corpus callosum to measure background values. To control variations in background illumination, the average of background density readings from the corpus callosum was subtracted from that of density readings of the striatum for each section. For each animal, the average of all sections was calculated separately before data were statistically processed.

### 2.5. Rotarod Test

We measured the ability of the animal to balance itself and remain on an accelerating rotarod (UgoBasile, Comerio, Italy) using two different experimental designs (fixed mode and accelerating mode) as previously described [25, 29]. To acclimate mice on the rotarod apparatus, animals were given a training session (10 rpm for 20 min), 7 consecutive days before MPTP injection. Animals that stayed on the rod without falling during training were selected and randomly divided into experimental groups. Seven days after the last MPTP treatment, animals receiving various treatment regimes were placed on the rotating rod and tested at 20 rpm for 20 min as fixed mode. On the next day, mice were tested at different progressively higher speeds on the rotarod apparatus that accelerates at a constant rate, from 4 to 40 rpm in 300 s as accelerating mode. The latency to fall was automatically recorded by magnetic trip plates.

### 2.6. FITC-Labeled Albumin Assay

As previously described [9], FITC-labeled albumin (MW = 69-70 kDa, Sigma, St. Louis, MO, USA) assay was performed for visualization of BBB leakage. Three days after MPTP or saline injections, mice were intranscardially perfused with Hank's Balanced Salt Solution containing heparin (10 U/mL) and then immediately by 5 mL FITC-labeled albumin (5 mg/mL, in 0.1 M phosphate-buffered saline (PBS) buffer) injected at a rate of 1.5 mL/minute. Brains were dissected from the skull, postfixed overnight in buffered 4% paraformaldehyde at 4°C. After fixation, the brains were cut into 30 *μ*m slices using a sliding microtome. Sections were mounted on gelatin-coated slides, and the FITC-labeled albumin contained vessels that were examined by confocal microscopy (Carl Zeiss). To determine the total area for FITC-labeled albumin leakage, 3 or 4 images of SN region were obtained, thresholded using Imag J, quantified, and normalized by value of PBS injected MMP-3^+/+^ mice.

### 2.7. Statistical Analysis

All values are expressed as mean ± SEM. Statistical significance (*P* < 0.05 for all analyses) was assessed by two-way ANOVA using Instat 3.05 (GraphPad Software, San Diego, CA, USA) followed by Bonferroni post hoc test.

## 3. Results 

### 3.1. MMP-3 Deficiency Protects Nigrostriatal DA Neurons from MPTP Neurotoxicity

**Figure 1:**
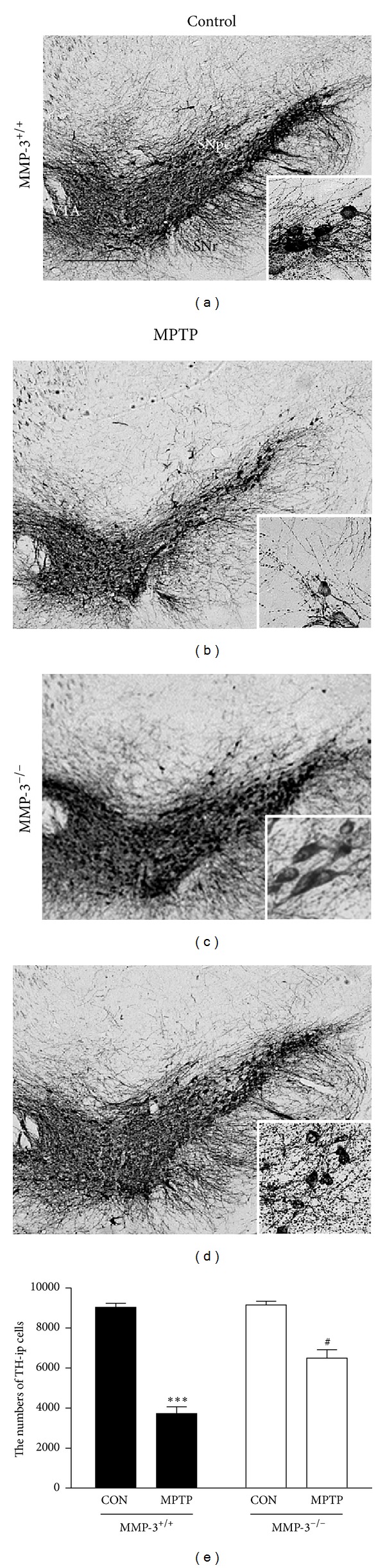
MPTP-induced neurotoxicity is attenuated in the SNpc of MMP-3^−/−^ mouse brain. (a) Animals (MMP-3^+/+^ or MMP-3^−/−^ mice) receiving PBS as a control (a and c) and MPTP (b and d) were sacrificed 7 days after the last MPTP injection. *Insets*, higher magnifications of (a–c). The brain tissues were cut into 30 *μ*m thick coronal sections using a sliding microtome and immunostained with an antibody against the DA neuronal marker TH. Scale bar, 300 *μ*m. (e) Bars represent the number of TH-ip neurons in the SN after indicated treatment in the absence (MMP-3^−/−^) or presence (MMP-3^+/+^) of MMP-3. Five to six animals were used for each experimental group. Two-way ANOVA with Bonferroni post hoc test (*F*(1,15) = 17.57, *P* < 0.001), ***P < 0.001, significantly different from PBS-injected MMP-3^+/+^ mice; ^#^ *P* < 0.05, significantly different from MPTP-injected MMP-3^+/+^ mice. SNpc, substantia nigra pars compacta; VTA, ventral tegmental area; SNr, substantia nigra reticulata.

**Figure 2:**
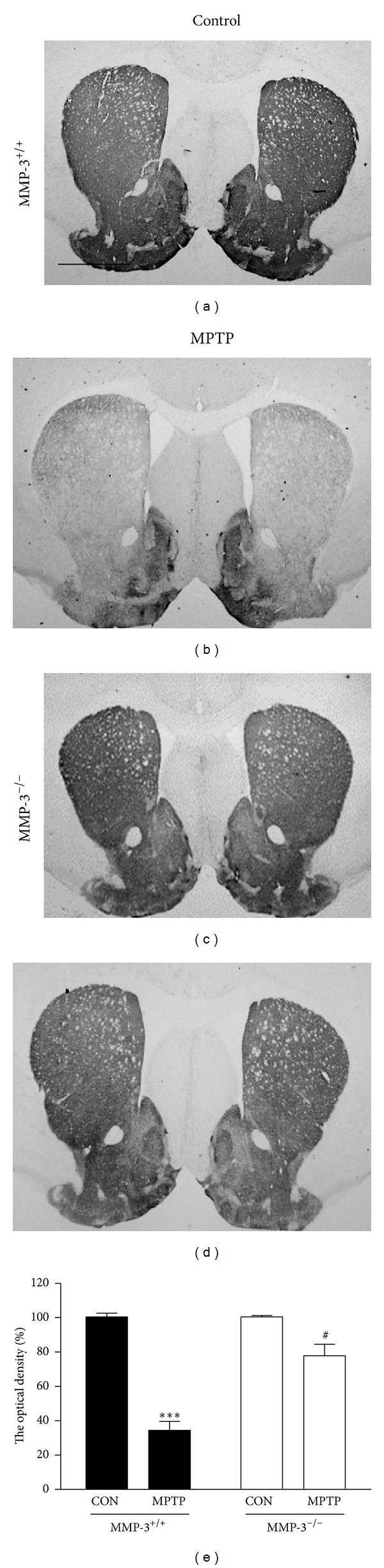
MPTP-induced neurotoxicity is attenuated in the striatum of MMP-3^−/−^ mouse brain. Striatal tissues obtained from the same animals as used in Figure 1 were immunostained with TH antibody for DA fibers. Animals were treated with PBS as a control (a and c) and MPTP (b and d). (e) Bars represent optical density of TH-ip fibers in the striatum. Scale bar, 250 *μ*m. Five to six animals were used for each experimental group. Two-way ANOVA with Bonferroni post hoc test (*F*(1,15) = 22.22, *P* < 0.001),  ***P < 0.001, significantly different from PBS-injected MMP-3^+/+^ mice; ^#^ *P* < 0.05, significantly different from MPTP-injected MMP-3^+/+^ mice.

Mice in each group (MMP-3^+/+^ or MMP-3^−/−^ mice) received four intraperitoneal injections of MPTP (20 mg/kg) or PBS (control) at 2-hour intervals. Seven days later, brains were removed, and sections were immunostained for TH to specifically detect DA neurons. Consistent with our recent reports [24–26], there was a significant loss of TH-immunopositive (ip) cell bodies in the SNpc (Figure 1(b)) and of TH-ip fibers in the STR (Figure 2(b)) at 7 days in MPTP-injected MMP-3^+/+^ mice compared with PBS-treated MMP-3^+/+^ control mice (Figures 1(a) and 2(a)). TH-ip cells in the SNpc and TH-ip fibers in the STR were quantified by stereological counts and densitometric analyses, respectively. The results showed that MPTP treatment reduced the number of TH-ip neurons by 59% (Figure 1(e), *P* < 0.001) and decreased the optical density (OD) of TH-ip fibers by 64% (Figure 2(e), *P* < 0.001) compared with PBS-treated MMP-3^+/+^ control mice.

In contrast to the above, the results of TH immunohistochemistry demonstrated that in MMP-3-deficient (MMP-3^−/−^) mice, the loss of TH-ip cell bodies in the SNpc (Figure 1(d)) and TH-ip fibers in the STR (Figure 2(d)) was significantly reduced compared to that seen in MPTP-treated MMP-3^+/+^ mice (Figures 1(b) and 2(b)). Stereological counts and densitometric analyses showed that the number of TH-ip neurons in the SNpc and the OD in the STR was higher by 30% (Figure 1(e); *P* < 0.05) and 31% (Figure 2(e); *P* < 0.05), respectively, in MMP-3^−/−^ mice compared to MMP-3^+/+^ mice. These data confirm our previous finding showing that MMP-3 participates in MPTP-induced neurotoxicity, resulting in the degeneration of nigrostriatal DA neurons *in vivo* [23].

### 3.2. MMP-3 Deficiency Improves Motor Behavior in MPTP Mice

**Figure 3:**
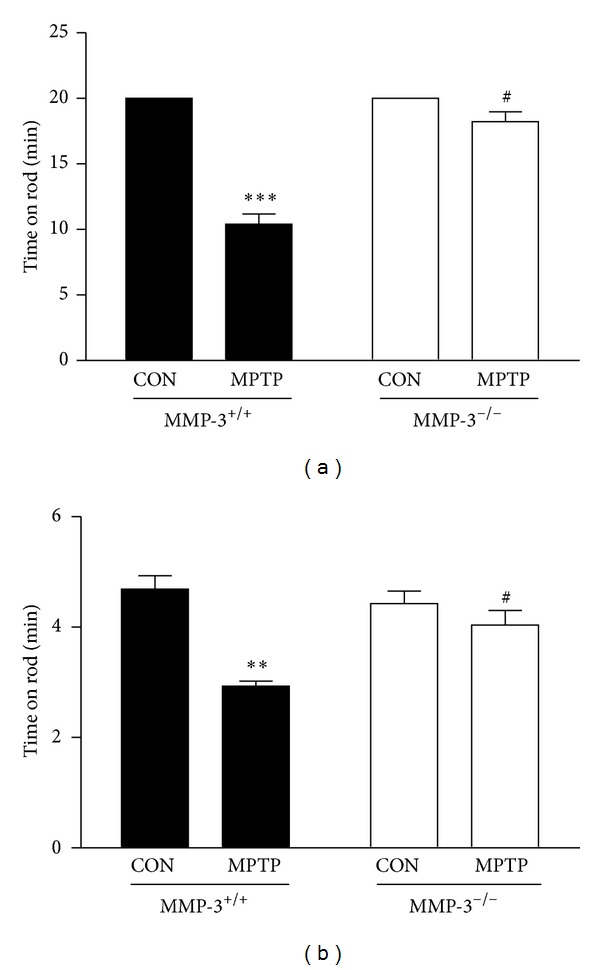
Effects of MMP-3 on motor performance in MPTP-treated mice. (a) After 7 days from MPTP injection, mice were subjected to rotating rod (20 rpm) for 20 min, and falling time was recorded. (b) Next day, mice were placed on an accelerating rotating rod, and the maximum time before the mouse fell from the rod was recorded. Five to six animals were used for each experimental group. Two way ANOVA with Bonferroni post hoc test, fixed mode; (*F*(1,15) = 45.67, *P* < 0.001), accelerating mode; (*F*(1,15) = 9.73, *P* < 0.01), ***P < 0.001 and  **P < 0.01, significantly different from PBS-injected MMP-3^+/+^ mice; ^#^ *P* < 0.05, significantly different from MPTP injected MMP-3^+/+^ mice.

We next used two different paradigms of rotarod performance to examine if MMP-3 affects MPTP-induced motor behavior [25, 29]. Animals receiving the different treatment regimens were evaluated 7 days after the last MPTP injection by measuring the fall time latency for the fixed mode (20 rpm for 20 min) and for the accelerating mode (4–40 rpm for 5 min) one day after carrying out the fixed mode evaluation. In MMP-3^+/+^ mice, the MPTP treatment decreased the sustained rotarod time in the fixed mode to 10.41 ± 0.78 min (representing a 48% decrease; *P* < 0.001; Figure 3(a)) and to 2.93 ± 0.91 min (representing a 37% decrease) in the accelerating mode (*P* < 0.001; Figure 3(b)), compared with control (PBS treatment). In contrast, this behavioral dysfunction in MPTP-treated MMP-3^+/+^ mice was partially reduced in MMP-3^−/−^ mice, which exhibited increased falling latencies of 18.52 ± 0.91 min (*P* < 0.05; Figure 3(a)) in the fixed mode and 4.03 ± 0.41 min (*P* < 0.05; Figure 3(b)) in the accelerating mode, respectively. The falling latency in both paradigms was not significantly different between PBS-injected MMP-3^−/−^ mice and their wild-type littermates.

### 3.3. MMP-3 Deficiency Prevents MPTP-Induced BBB Damage *In Vivo*

**Figure 4:**
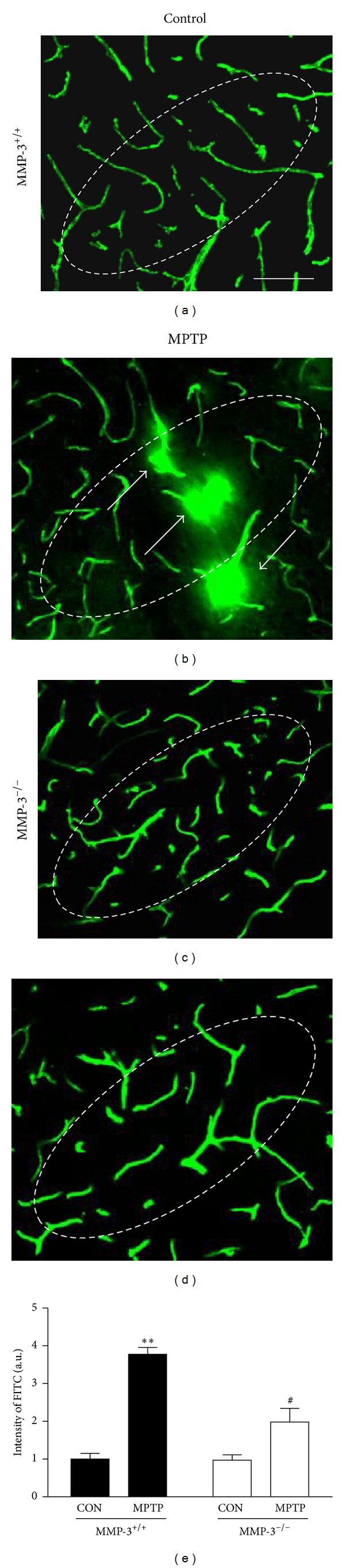
MPTP-induced BBB breakage is prevented in MMP-3^−/−^ mice. At 3 days after MPTP injection in the absence (MMP-3^−/−^; (c and d)) or presence (MMP-3^+/+^; (a and b)) of MMP-3, FITC-linked albumin was administered to detect for brain vascular permeability. (e) Bars represent the FITC-labeled albumin-positive area in the SNpc, respectively. Four or five animals were used for each experimental group. Whole values are normalized by PBS-injected MMP-3^+/+^ mice. Two-way ANOVA with Bonferroni post hoc test (*F*(1,15) = 10.19, *P* < 0.001). **P < 0.01, significantly different from PBS-injected MMP-3^+/+^ mice;  ^#^ *P* < 0.05, significantly different from MPTP-injected MMP-3^+/+^ mice. Arrows indicate FITC-linked albumin leakage, indicating extravasations of FITC-labeled albumin into the brain. Dotted lines indicate the SNpc, where DA neurons were degenerating after MPTP injection. Scale bar, 400 *μ*m.

Increased BBB permeability has been observed in the midbrains of PD patients [6, 30]. In this regard, several *in vivo* studies have also demonstrated that increased BBB permeability can play an important role in the induction of DA neuronal death in the MPTP mouse model of PD [8, 31]. Thus, we next investigated whether MMP-3 affects MPTP-induced BBB disruption by detecting FITC-labeled albumin in the brain three days after the last MPTP treatment. In PBS-injected MMP-3^+/+^ (Figure 4(a)) and MMP-3^−/−^ mice (Figure 4(c)), FITC-labeled albumin was confined to the blood vessels of the SN *in vivo*, indicating that the BBB was intact. However, in the SN of MPTP-treated MMP-3^+/+^ mice, the diffusion of FITC-labeled albumin into the brain from multiple blood vessels was clearly evident (*P* < 0.01; Figures 4(b) and 4(e)). In contrast, the vascular diffusion of FITC-labeled albumin was not as clear-cut in MPTP-treated MMP-3^−/−^ mice (*P* < 0.05; Figures 4(d) and 4(e)). These results suggest that MMP-3 could be involved in an MPTP-induced damage to the BBB.

### 3.4. MMP-3 Deficiency Inhibits Microglial Activation and the Infiltration of T Cells into the SN *In Vivo*

**Figure 5:**
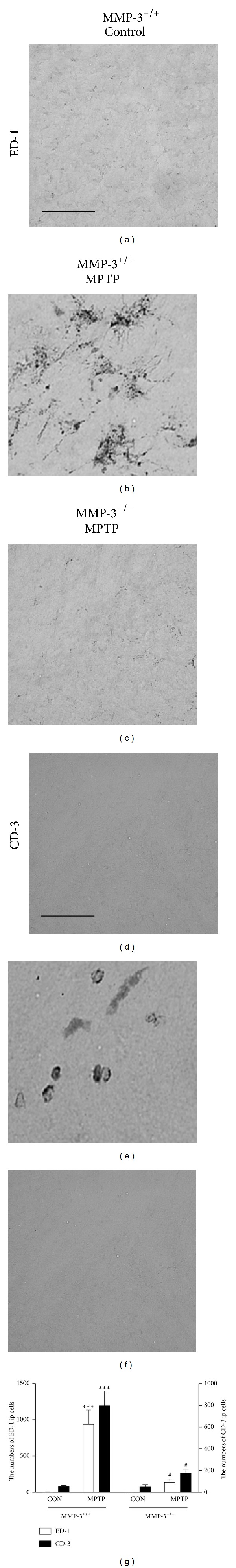
MPTP-induced increases in ED-1 and CD-3-ip cells are suppressed in the SNpc of MMP-3^−/−^ mouse brain. ((a–f)) Sections adjacent to those used for FITC-linked albumin staining were examined for ED-1 or CD-3 immunostaining. (g) Bars represent the numbers of ED-1 or CD-3-ip cells in the SNpc, respectively. Four or five animals were used for each experimental group. Dotted lines indicate the SNpc. Scale bar, 100 *μ*m. Two-way ANOVA with Bonferroni post hoc test, ED-1-ip cells; (*F*(1,15) = 45.67, *P* < 0.001), CD-3-ip cells; (*F*(1,15) = 13.72, *P* < 0.01), ***P < 0.001, significantly different from PBS-injected MMP-3^+/+^ mice; ^#^ *P* < 0.05, significantly different from MPTP-injected MMP-3^+/+^ mice.

Accumulating evidence, including that from our group, suggests that activated microglia play an important role in DA neuronal cell death in the MPTP mouse model [3, 32]. Accordingly, we next examined if the resistance to MPTP neurotoxicity exhibited by MMP-3-deficient mice could result from the inhibition of microglial activation in the SN. Three days after the last MPTP injection, sections of brain tissue from mice were immunostained with ED-1 antibody, a marker for activated microglia. In contrast to the SN of control mice (MMP-3^+/+^ mice) treated with PBS (Figure 5(a)), where relatively few ED-1-ip microglia were observed, the SN of MPTP-treated MMP-3^+/+^ mice contained numerous ED-1-ip cells. The majority of ED-1-ip microglia in the MPTP-treated SN of MMP-3^+/+^ mice displayed an activated morphology, including larger cell bodies with short and thick processes or no processes at all (Figure 5(b)). In contrast, the number of ED-1-ip-activated microglia was clearly decreased in the SN of MPTP-treated MMP-3^−/−^ mice (Figures 5(c) and 5(g)). 

 Recent studies have shown that the migration of peripheral T lymphocytes within the CNS is associated with DA neuronal death in the SN of PD patients and of MPTP-treated mice [5, 33]. Accordingly, we examined whether a deficiency of MMP-3 inhibits the infiltration of T lymphocytes. As described previously [32], the MPTP-induced infiltration of T cells was visualized by CD-3 immunostaining of sections adjacent to those used for ED-1 immunostaining. Compared with the SN of PBS-treated MMP-3^+/+^ mice (Figure 5(d)), a 14-fold increase in the number of CD-3-ip cells in the SN of MPTP-treated MMP-3^+/+^ mice was seen three days after the last MPTP injection (*P* < 0.001; Figures 5(e) and 5(g)). By comparison, the number of CD-3-ip cells in the SN of MMP-3^−/−^ mice was 79% lower than that in the MMP-3^+/+^ mice (*P* < 0.05; Figures 5(f) and 5(g)). Stereological cell counting showed that the numbers of ED-1 and CD-3-ip cells in the SN were similar between PBS-injected MMP-3^−/−^ mice and their wild-type littermates (Figure 5(g)).

## 4. Discussion

Accumulating evidence suggests that MMP-3 is associated with DA neuronal death and neuroinflammation and as a consequence is involved in the pathogenesis of PD [10, 34]. Several studies have previously shown that MMP-3 plays an important role in caspase signaling under ER stress [35] or BH-4- [36] induced apoptotic DA neuronal death and is involved in the induction of reactive oxygen species (ROS) and proinflammatory cytokine production in activated microglia [15]. The pharmacological inhibition of MMP-3 recovers motor deficits and suppresses microglia activation in MPTP-treated mice [37]. We have shown here, for the first time, that in addition to degeneration of DA neurons and microglial activation, MMP-3 participates in the impairment of BBB integrity and T-leukocyte infiltration into the SN of MPTP-treated MMP-3^+/+^ mice.

Neuroinflammation can be produced by many factors, including activated microglia, BBB disruption, and infiltration of peripheral immune cells into the brain and as such plays a critical role in the CNS immune system [38]. Intrinsic immune mechanisms perform neuroprotective and supportive functions in the normal CNS; however, under neuropathological conditions, neuroinflammation may be triggered by transient initiation factors such as neuronal damage, thereby contributing to irreversible DA neuronal death in the SN and locomotor deficits [39].

The pathogenesis of PD has been linked increasingly to neuroinflammation and BBB impairment [40–42]. Neuroinflammation was found to exert harmful effects on BBB integrity [43], and BBB leakage has been demonstrated in PD patients [6, 30] and in MPTP- or 6-OHDA-treated animal models of PD [9, 44]. Because the BBB helps regulate and protect the microenvironment of the brain and its disruption results in the loss of DA neurons [45], it has been hypothesized that BBB dysfunction may account for, at least in part the degeneration of DA neurons in PD [46, 47].

The BBB, which is comprised of neurovascular units such as endothelial cells, pericytes, neurons, and astrocyte end-feet, restricts the entry of plasma components, blood cells, and leukocytes into the brain. When these infiltrate into the brain parenchyma due to neurodegenerative processes or neuroinflammation, neurotoxic substances can be produced that cause neuronal dysfunction and loss [43]. We recently demonstrated in LPS-treated rats and MPTP-treated mice that compromising the integrity of the BBB contributes to the degeneration of nigrostriatal DA neurons in the SN [32]. The present data show that MPTP increases the infiltration of FITC-labeled albumin from blood vessels into the SN and that genetic deletion of the MMP-3 (i.e., MMP-3^−/−^ mice) likely attenuates the MPTP-induced damage to the BBB and subsequent impact on the SN.

Glial cell activation is one of the major contributors to neuroinflammation and is implicated in the pathogenesis and progression of PD [32, 38]. In particular, activated microglia can release harmful substances such as proinflammatory cytokines, ROS, and reactive nitrogen species which subsequently cause neuronal loss and dysfunction in a range of neurodegenerative diseases [3]. Several studies have demonstrated that reactive microglia expressing proinflammatory mediators are present in the midbrains of PD patients [48–50], while increasing evidence, including our own [24–26], has shown that activated microglia contribute to DA neuronal cell death through NADPH- [50] and MPO- [48] mediated oxidative stress and production of proinflammatory molecules (iNOS, TNF-*α*, and IL-1*β*) [51] in the MPTP mouse model of PD. Recently, we reported that the number of CD-11b- and Iba-1-ip-activated microglia was correlated with ED-1-ip microglia/macrophage phagocytotic activity in the SN of MPTP-treated mice [26]. With respect to that finding, the present study shows that MPTP significantly upregulated the expression of ED-1-ip microglia in the SN. In contrast, the increase in number of ED-1-ip cells was dramatically attenuated in the MPTP-treated SN of MMP-3 knockout (MMP-3^−/−^) mice. These data support the hypothesis that MMP-3 has the capacity to induce microglial activation, resulting in the degeneration of DA neurons.

In addition to microglial activation, the infiltration of T leukocytes may be involved in nigrostriatal DA neuronal death. Several lines of evidence highlight the presence of infiltrating T cells (CD-4- or CD-8-ip cells) in the SN of PD patients [5] and MPTP-treated mice [5, 33]. Brochard and colleagues also showed the CD-4- but not CD-8-mediated degeneration of DA neurons in the SN of MPTP-treated mice [5]. Depboylu and colleagues recently showed that infiltrating CD-3-ip T lymphocytes, representing both CD-4-ip and CD-8-ip cells, are implicated in the regulation of the adaptive immune system through crosstalk with microglia and/or macrophages in the SN in the MPTP mouse model of PD [33]. Moreover, CD-4-ip T cells mediated (via the Fas/Fas ligand pathway) the detrimental effects on DA neurons in the SN of MPTP-treated mice [5]. In this way, the data in this report show that MPTP increases the number of infiltrating CD-3-ip T cells in the SN of MMP-3^+/+^ control mice; this number was significantly reduced in the SN of MPTP-treated MMP-3^−/−^ mice, eventually leading to the improved survival of DA neurons. These data suggest that MMP-3-induced T-cell infiltration contributes to MMP-3's neurotoxic effect on DA neurons in the SN. 

The predominant behavioral change in the MPTP-treated mice was the reduced latency to fall from the rotarod apparatus, thereby reflecting diminished coordination and balance [24–26]. The two different designs of rotarod performance (fixed mode and accelerating mode) indicate that nigrostriatal DA neuron loss is well correlated with motor dysfunction on this apparatus. Consistent with our recent data [24, 26], MPTP reduced the latency to fall from the rotarod apparatus in MMP-3^+/+^ control mice. This behavioral dysfunction was partially improved in MPTP-treated MMP-3^−/−^ mice. These behavioral effects of MMP-3 deletion on the lesioned nigrostriatal DA system, together with the knowledge that the genetic ablation of MMP-3 prevents microglial activation, infiltration of T leukocyte, and BBB disruption, suggest that MMP-3 could be a useful therapeutic target for treating PD and other neurodegenerative disorders involving neuroinflammation and compromised BBB integrity.

## 5. Conclusion

The present study shows that matrix metalloproteinase-3 (MMP-3) participates in degeneration of nigrostriatal dopaminergic neurons in the MPTP model of Parkinson's disease by neuroinflammation-mediated BBB disruption and infiltration of T leukocytes. To our knowledge, this is the first study to demonstrate that neurotoxic effects of MMP-3 in the MPTP-treated SN are associated with the ability of MMP-3 to increase BBB damages, microglia/macrophage phagocytosis, and infiltration of T leukocytes, suggesting that MMP-3 can be novel therapeutic target for PD and other disorders associated with BBB integrity and neuroinflammation.

## References

[1] Savitt JM, Dawson VL, Dawson TM (2006). Diagnosis and treatment of Parkinson disease: molecules to medicine. *Journal of Clinical Investigation*.

[2] Appel SH (2009). CD4^+^ T cells mediate cytotoxicity in neurodegenerative diseases. *Journal of Clinical Investigation*.

[3] Block ML, Zecca L, Hong J-S (2007). Microglia-mediated neurotoxicity: uncovering the molecular mechanisms. *Nature Reviews Neuroscience*.

[4] Weiss N, Miller F, Cazaubon S, Couraud P-O (2009). The blood-brain barrier in brain homeostasis and neurological diseases. *Biochimica et Biophysica Acta*.

[5] Brochard V, Combadière B, Prigent A (2009). Infiltration of CD4^+^ lymphocytes into the brain contributes to neurodegeneration in a mouse model of Parkinson disease. *Journal of Clinical Investigation*.

[6] Pisani V, Stefani A, Pierantozzi M (2012). Increased blood-cerebrospinal fluid transfer of albumin in advanced Parkinson's disease. *Journal of Neuroinflammation*.

[7] Faucheux BA, Bonnet A-M, Agid Y, Hirsch EC (1999). Blood vessels change in the mesencephalon of patients with Parkinson’s disease. *The Lancet*.

[8] Chao YX, He BP, Tay SSW (2009). Mesenchymal stem cell transplantation attenuates blood brain barrier damage and neuroinflammation and protects dopaminergic neurons against MPTP toxicity in the substantia nigra in a model of Parkinson’s disease. *Journal of Neuroimmunology*.

[9] Zhao C, Ling Z, Newman MB, Bhatia A, Carvey PM (2007). TNF-*α* knockout and minocycline treatment attenuates blood-brain barrier leakage in MPTP-treated mice. *Neurobiology of Disease*.

[10] Kim E-M, Hwang O (2011). Role of matrix metalloproteinase-3 in neurodegeneration. *Journal of Neurochemistry*.

[11] van Hove I, Lemmens K, van de Velde S, Verslegers M, Moons L (2012). Matrix metalloproteinase-3 in the central nervous system: a look on the bright side. *Journal of Neurochemistry*.

[12] Gonthier B, Nasarre C, Roth L (2007). Functional interaction between matrix metalloproteinase-3 and semaphorin-3C during cortical axonal growth and guidance. *Cerebral Cortex*.

[13] van Hove I, Verslegers M, Buyens T (2012). An aberrant cerebellar development in mice lacking matrix metalloproteinase-3. *Molecular Neurobiology*.

[14] Meighan SE, Meighan PC, Choudhury P (2006). Effects of extracellular matrix-degrading proteases matrix metalloproteinases 3 and 9 on spatial learning and synaptic plasticity. *Journal of Neurochemistry*.

[15] Kim YS, Kim SS, Cho JJ (2005). Matrix metalloproteinase-3: a novel signaling proteinase from apoptotic neuronal cells that activates microglia. *Journal of Neuroscience*.

[16] Gurney KJ, Estrada EY, Rosenberg GA (2006). Blood-brain barrier disruption by stromelysin-1 facilitates neutrophil infiltration in neuroinflammation. *Neurobiology of Disease*.

[17] Choi DH, Kim E-M, Son HJ (2008). A novel intracellular role of matrix metalloproteinase-3 during apoptosis of dopaminergic cells. *Journal of Neurochemistry*.

[18] Kim ST, Kim E-M, Choi JH (2010). Matrix metalloproteinase-3 contributes to vulnerability of the nigral dopaminergic neurons. *Neurochemistry International*.

[19] Sung JY, Park SM, Lee C-H (2005). Proteolytic cleavage of extracellular secreted *α*-synuclein via matrix metalloproteinases. *Journal of Biological Chemistry*.

[20] Choi D-H, Kim Y-J, Kim Y-G, Joh TH, Beal MF, Kim Y-S (2011). Role of matrix metalloproteinase 3-mediated *α*-synuclein cleavage in dopaminergic cell death. *Journal of Biological Chemistry*.

[21] Kim S, Moon M, Park S (2009). Exendin-4 protects dopaminergic neurons by inhibition of microglial activation and matrix metalloproteinase-3 expression in an animal model of Parkinson’s disease. *Journal of Endocrinology*.

[22] Moon M, Kim HG, Hwang L (2009). Neuroprotective effect of ghrelin in the 1-methyl-4-phenyl-1,2,3,6-tetrahydropyridine mouse model of parkinson’s disease by blocking microglial activation. *Neurotoxicity Research*.

[23] Kim YS, Choi DH, Block ML (2007). A pivotal role of matrix metalloproteinase-3 activity in dopaminergic neuronal degeneration via microglial activation. *The FASEB Journal*.

[24] Chung YC, Bok E, Huh SH (2011). Cannabinoid receptor type 1 protects nigrostriatal dopaminergic neurons against MPTP neurotoxicity by inhibiting microglial activation. *Journal of Immunology*.

[25] Chung YC, Kim SR, Jin BK (2010). Paroxetine prevents loss of nigrostriatal dopaminergic neurons by inhibiting brain inflammation and oxidative stress in an experimental model of Parkinson’s disease. *Journal of Immunology*.

[26] Huh SH, Chung YC, Piao Y (2011). Ethyl pyruvate rescues nigrostriatal dopaminergic neurons by regulating glial activation in a mouse model of Parkinson’s disease. *Journal of Immunology*.

[27] Chung YC, Kim SR, Park J-Y (2011). Fluoxetine prevents MPTP-induced loss of dopaminergic neurons by inhibiting microglial activation. *Neuropharmacology*.

[28] West MJ, Slomianka L, Gundersen HJG (1991). Unbiased stereological estimation of the total number of neurons in the subdivisions of the rat hippocampus using the optical fractionator. *Anatomical Record*.

[29] Chagniel L, Robitaille C, Lacharité-Mueller C, Bureau G, Cyr M (2012). Partial dopamine depletion in MPTP-treated mice differentially altered motor skill learning and action control. *Behavioural Brain Research*.

[30] Kortekaas R, Leenders KL, van Oostrom JCH (2005). Blood-brain barrier dysfunction in Parkinsonian midbrain *in vivo*. *Annals of Neurology*.

[31] Chen X, Lan X, Roche I, Liu R, Geiger JD (2008). Caffeine protects against MPTP-induced blood-brain barrier dysfunction in mouse striatum. *Journal of Neurochemistry*.

[32] Chung YC, Ko HW, Bok E (2010). The role of neuroinflammation on the pathogenesis of Parkinson’s disease. *BMB Reports*.

[33] Depboylu C, Stricker S, Ghobril JP, Oertel WH, Priller J, ِglinger GU H (2012). Brain-resident microglia predominate over infiltrating myeloid cells in activation, phagocytosis and interaction with T-lymphocytes in the MPTP mouse model of Parkinson disease. *Experimental Neurology*.

[34] Kim YS, Joh TH (2006). Microglia, major player in the brain inflammation: their roles in the pathogenesis of Parkinson’s disease. *Experimental and Molecular Medicine*.

[35] Kim E-M, Shin E-J, Choi JH (2010). Matrix metalloproteinase-3 is increased and participates in neuronal apoptotic signaling downstream of caspase-12 during endoplasmic reticulum stress. *Journal of Biological Chemistry*.

[36] Cho Y, Son HJ, Kim E-M (2009). Doxycycline is neuroprotective against nigral dopaminergic degeneration by a dual mechanism involving MMP-3. *Neurotoxicity Research*.

[37] Son HJ, Lee JA, Shin N (2012). A novel compound PTIQ protects the nigral dopaminergic neurones in an animal model of Parkinson’s disease induced by MPTP. *British Journal of Pharmacology*.

[38] Hirsch EC, Hunot S (2009). Neuroinflammation in Parkinson’s disease: a target for neuroprotection?. *The Lancet Neurology*.

[39] Tansey MG, Goldberg MS (2010). Neuroinflammation in Parkinson’s disease: its role in neuronal death and implications for therapeutic intervention. *Neurobiology of Disease*.

[40] Abbott NJ, Rönnbäck L, Hansson E (2006). Astrocyte-endothelial interactions at the blood-brain barrier. *Nature Reviews Neuroscience*.

[41] Hawkins BT, Davis TP (2005). The blood-brain barrier/neurovascular unit in health and disease. *Pharmacological Reviews*.

[42] Hunot S, Hirsch EC, Isacson I (2003). Neuroinflammatory processes in Parkinson’s disease. *Annals of Neurology*.

[43] Zlokovic BV (2008). The blood-brain barrier in health and chronic neurodegenerative disorders. *Neuron*.

[44] Carvey PM, Zhao CH, Hendey B (2005). 6-Hydroxydopamine-induced alterations in blood-brain barrier permeability. *European Journal of Neuroscience*.

[45] Rite I, Machado A, Cano J, Venero JL (2007). Blood-brain barrier disruption induces *in vivo* degeneration of nigral dopaminergic neurons. *Journal of Neurochemistry*.

[46] Desai BS, Monahan AJ, Carvey PM, Hendey B (2007). Blood-brain barrier pathology in Alzheimer’s and Parkinson’s disease: implications for drug therapy. *Cell Transplantation*.

[47] Monahan AJ, Warren M, Carvey PM (2008). Neuroinflammation and peripheral immune infiltration in Parkinson’s disease: an autoimmune hypothesis. *Cell Transplantation*.

[48] Choi D-K, Pennathur S, Perier C (2005). Ablation of the inflammatory enzyme myeloperoxidase mitigates features of Parkinson’s disease in mice. *Journal of Neuroscience*.

[49] Nagatsu T, Sawada M (2007). Biochemistry of postmortem brains in Parkinson’s disease: historical overview and future prospects. *Journal of Neural Transmission*.

[50] Wu D-C, Teismann P, Tieu K (2003). NADPH oxidase mediates oxidative stress in the 1-methyl-4-phenyl-1,2,3,6-tetrahydropyridine model of Parkinson’s disease. *Proceedings of the National Academy of Sciences of the United States of America*.

[51] Wu DC, Jackson-Lewis V, Vila M (2002). Blockade of microglial activation is neuroprotective in the 1-methyl-4-phenyl-1,2,3,6-tetrahydropyridine mouse model of Parkinson disease. *Journal of Neuroscience*.

